# Myeloid Cell-Specific Lipin-1 Deficiency Stimulates Endocrine Adiponectin-FGF15 Axis and Ameliorates Ethanol-Induced Liver Injury in Mice

**DOI:** 10.1038/srep34117

**Published:** 2016-09-26

**Authors:** Jiayou Wang, Chunki Kim, Alvin Jogasuria, Yoonhee Han, Xudong Hu, Jiashin Wu, Hong Shen, Roman Chrast, Brian N. Finck, Min You

**Affiliations:** 1 Department of Pharmaceutical Sciences, College of Pharmacy, Northeast Ohio Medical University, Rootstown, USA; 2 Department of Biology, School of Basic Medical Science, Shanghai University of Traditional Chinese Medicine, Shanghai, China; 3 Department of Liver Diseases, Guangdong Hospital of Traditional Chinese Medicine in Zhuhai, Zhuhai, China; 4 Department of Neuroscience, Karolinska Institute, Stockholm, Sweden; 5 Division of Geriatrics and Nutritional Science, Washington University School of Medicine, St. Louis, USA

## Abstract

Lipin-1 is a phosphatidate phosphohydrolase (PAP) required for the generation of diacylglycerol during glycerolipid synthesis, and exhibits dual functions in the regulation of lipid metabolism. Lipin-1 has been implicated in the pathogenesis of alcoholic liver disease (ALD). In the present study, we assessed lipin-1 function in myeloid cells in ALD using a myeloid cell-specific lipin-1 knockout (mLipin-1KO) mouse model. Utilizing the Gao-binge ethanol feeding protocol, matched mLipin-1KO mice and littermate loxP control (WT) mice were pair-fed with either an ethanol-containing diet or an ethanol-free diet (control). Surprisingly, deletion of lipin-1 in myeloid cells dramatically attenuated liver inflammatory responses and ameliorated liver injury that would normally occur following the ethanol feeding protocol, but slightly exacerbated the ethanol-induced steatosis in mice. Mechanistically, myeloid cell-specific lipin-1 deficiency concomitantly increased the fat-derived adiponectin and ileum-derived fibroblast growth factor (FGF) 15. In concordance with concerted elevation of circulating adiponectin and FGF15, myeloid cell-specific lipin-1 deficiency diminished hepatic nuclear factor kappa B (NF-κB) activity, limited liver inflammatory responses, normalized serum levels of bile acids, and protected mice from liver damage after ethanol challenge. Our novel data demonstrate that myeloid cell-specific deletion of lipin-1 ameliorated inflammation and alcoholic hepatitis in mice via activation of endocrine adiponectin-FGF15 signaling.

Heavy chronic alcohol consumption, especially when combined with binging, is associated with development of alcoholic liver disease (ALD)^1^. Elevated hepatic inflammation and liver injury are hallmarks of clinical patients with ALD^1^,^2^. However, pathogenic mechanisms of ethanol-induced inflammation and alcoholic hepatitis are still largely unclear. To date, patients with alcoholic hepatitis lack effective treatments.

Lipin-1, a mammalian Mg^2+^-dependent phosphatidate phosphohydrolase (PAP), is a bi-functional molecule that regulates lipid metabolism at multiple levels^3^. Lipin-1 converts phosphatidate into diacylglycerol through its PAP activity and contributes to triglyceride (TG) synthesis. Lipin-1 also translocates into nucleus and interacts with DNA-bound transcription factors such as proliferator-activated receptor α, PPARγ coactivator 1α, hepatic nuclear receptor 4α, and sterol-response element binding protein 1 to regulate their activities^4^,^5^.

While the role of lipin-1 in regulating lipid metabolism is established, accumulating evidences suggest complex and controversial functional roles of lipin-1 in regulating inflammation process^6^,^7^,^8^,^9^,^10^,^11^,^12^,^13^. Several studies have demonstrated that lipin-1 has potent anti-inflammatory properties^6^,^7^,^9^,^10^. In adipocytes, lipin-1 inhibited nuclear factor activated T cells c4 transcriptional activity in a PAP independent manner, which, in turn, inhibited production of a panel of proinflammatory cytokines^6^. Inhibition of lipin-1 gene expression also increased monocyte chemoattractant protein-1, a regulator of adipose inflammation^7^. Furthermore, hepatocyte-specific lipin-1 ablation significantly increased the mRNA levels of several important pro-inflammatory cytokines, such as tumor necrosis factor-alpha (TNF-α), interleukin-1beta (IL-1β), lipocalin-2 (Lcn-2) and serum amyloid A-1 (Saa-1), in mice^9^. Conversely, other studies also demonstrated lipin-1 as a major contributor to macrophage proinflammatory activation^11^,^12^,^13^. For instance, upon Toll-Like-Receptor (TLR) stimulation of macrophages from humans or lipin-1-deficient mice, the generation of proinflammatory cytokines known to involve in inflammatory process was reduced^12^. This suggests that lipin-1 acts as a proinflammatory mediator downstream of TLR signaling and during the development of inflammatory processes in macrophage. Intriguingly, lipin-1 contributed to lipid droplet formation in response to fatty acid loading in cultured mouse and human macrophages. The contribution of lipin-1 to glycerophospholipid synthesis was necessary for new membrane generation during B cell differentiation, implying that lipin-1 might also link lipid synthesis pathways that contributed to inflammation in macrophages^7^,^13^.

Emerging evidences suggested that ethanol-mediated alterations in lipin-1 functions contribute to abnormalities in the hepatic lipid metabolism and inflammatory processes associated with ALD in rodents and human^9^,^10^,^14^,^15^,^16^,^17^,^18^. We discovered that hepatocyte-specific lipin-1 ablation exacerbated inflammation and aggravated the development and progression of experimental steatohepatitis in mice after ethanol challenge, suggesting that hepatocyte-specific lipin-1 exerts anti-inflammatory properties in alcoholic steatohepatitis^9^. However, it remains unknown whether and how myeloid cell-specific lipin-1 influences alcoholic steatosis, inflammatory conditions and liver injury *in vivo*.

In the present study, to determine the effects of loss of lipin-1 in myeloid cells on the development and progression of alcoholic steatohepatitis, we generated a myeloid cell-specific lipin-1 knockout mouse (mLipin-1KO) model utilizing a loxP/Cre recombinase system. We demonstrated that ablation of myeloid cell-specific lipin-1 selectively ameliorated hepatic inflammation but not steatosis in mice after ethanol administration. We also provided novel evidence demonstrating that the protective effects of myeloid cell-specific lipin-1 deficiency against inflammation and liver injury in the ethanol-exposed mice were mediated, at least in part, through coordinated elevation and stimulation of two prominent endocrine hormones, adipose-derived adiponectin and gut-derived fibroblast growth factor (FGF) 15, and their hepatic signaling.

## Results

### Myeloid cell-specific lipin-1 deficiency protected mice from ethanol-induced liver injury but mildly aggravated steatosis

Our mLipin-1KO mice, generated with the loxP/Cre recombinase system, were viable and phenotypically normal under a chow diet^9^,^19^. The role of myeloid cell-specific lipin-1 deficiency on alcoholic steatohepatitis was investigated by pair-feeding wild-type (WT) and mLipin-1KO mice utilizing a Gao-binge ethanol feeding protocol^20^.

**Figure 1:**
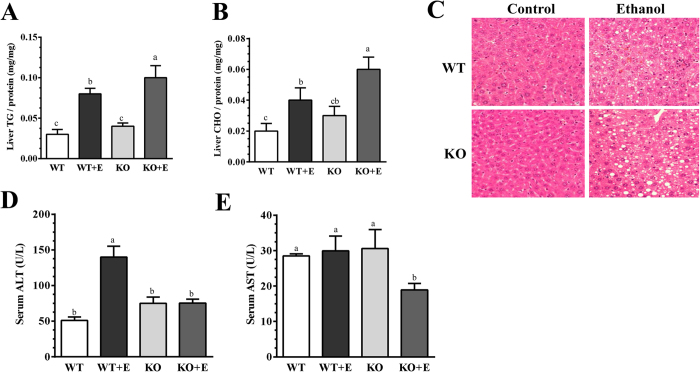
Myeloid cell-specific lipin-1 deficiency alleviated experimental alcoholic steatohepatitis in mice. Wild-type (WT) or mLipin-1KO (KO) mice were pair-fed either a control diet or an ethanol-containing diet for 10 days followed by single gavage of ethanol. (**A**) Liver triglyceride (TG) levels. (**B**) Liver cholesterol (CHO) levels. (**C**) Immunohistochemical staining for Hematoxylin and eosin (H&E) (original magnification ×40) of liver sections (**D**) Serum ALT. (**E**) Serum AST. Results are expressed as means ± SEM (n = 4–6 mice). Means without a common letter differ, *P* < 0.05.

As expected, ethanol feeding to WT mice significantly increased the levels of hepatic TGs and cholesterol compared with their pair-fed WT controls (Fig. 1A,B). The increases in hepatic TGs and cholesterol were more pronounced in the ethanol-fed mLipin-1LKO mice than their corresponding pair-fed WT mice (Fig. 1A,B). H&E staining analysis confirmed that ethanol administration slightly augmented accumulation of lipid droplets in livers of mLipin-1LKO mice compared to the other three groups (Fig. 1C).

Serum alanine transaminase (ALT), an indicator of liver damage, was significantly elevated in the ethanol-fed WT compared to WT control mice (Fig. 1D). Surprisingly, the ethanol-feeding-associated elevation of serum ALT was significantly reduced in the mLipin-1KO mice compared to WT mice (Fig. 1D). Concordantly, the serum levels of aspartate aminotransferase (AST) in the mLipin-1KO mice were the lowest among all groups (Fig. 1E).

Collectively, the data demonstrated that myeloid cell-specific lipin-1 deficiency ameliorated liver damaged but slightly exacerbated steatosis in mice after ethanol administration.

### Myeloid cell-specific lipin-1 deficiency ameliorated hepatic inflammation by ethanol in mice

**Figure 2:**
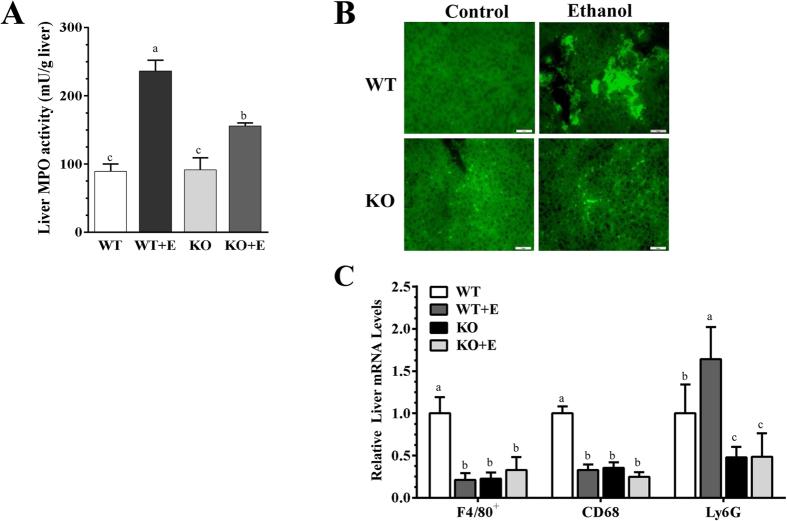
Myeloid cell-specific lipin-1 deficiency alleviated experimental alcoholic steatohepatitis in mice. Wild-type (WT) or mLipin-1KO (KO) mice were pair-fed either a control diet or an ethanol-containing diet for 10 days followed by single gavage of ethanol. (**A**) Liver MPO activity. (**B**) Immunohistochemical staining for MPO (original magnification ×40) of liver sections. (**C**) Relative liver mRNA levels of F4/80^+^, CD68, and Ly6G were analyzed by real-time PCR (qRT-PCR). Results are expressed as means ± SEM (n = 4–6 mice). Means without a common letter differ, *P* < 0.05.

Neutrophilic inflammation contributes to the ethanol-induced hepatic dysfunction and injury^21^. As illustrated in Fig. 2A, hepatic myeloperoxidase (MPO) activity was significantly increased in both the ethanol-fed WT mice and ethanol-fed mLipin-1KO mice compared to WT controls. However, in comparison to the ethanol-fed WT mice, ethanol-fed mLipin-1KO mice displayed significantly lower levels of hepatic MPO activity (Fig. 2A). Examination of liver histology with MPO staining revealed that neutrophil infiltration was markedly higher in livers from the ethanol-fed WT mice compared to WT controls (Fig. 2B). However, fewer MPO^+^ neutrophils infiltrated livers of the ethanol-fed mLipin-1KO mice compared with ethanol-fed WT mice (Fig. 2B). Accordingly, while ethanol feeding to WT mice significantly increased hepatic gene expression of a neutrophil marker, Ly6G, Ly6G mRNA levels were substantially reduced in the mLipin-1KO mice fed either with or without ethanol compared to WT controls, confirming that myeloid cell-specific lipin-1 deficiency attenuated hepatic neutrophilic inflammation in the ethanol-administrated mice (Fig. 2C).

**Figure 3:**
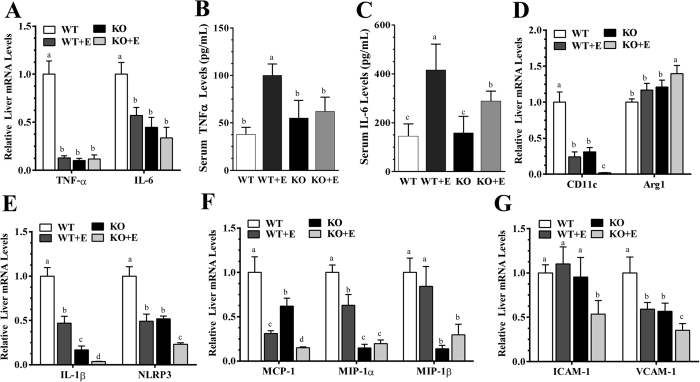
Myeloid cell-specific lipin-1 deficiency ameliorated ethanol-induced hepatic inflammation in mice. Wild-type (WT) or mLipin-1KO (KO) mice were pair-fed either a control diet or an ethanol-containing diet for 10 days followed by single gavage of ethanol. (**A**) Relative liver mRNA levels of TNF-α and IL-6. (**B**) Serum TNF-α levels. (**C**) Serum IL-6 levels. (**D**) Relative hepatic mRNA levels of CD11c and Arg1. (**E**) Relative hepatic mRNA levels of IL-1β and NLRP3. (**F**) Relative hepatic mRNA levels of MCP-1, MIP-1α and MIP-1β. (**G**) Relative hepatic mRNA levels of ICAM-1 and VCAM-1. Results are expressed as means ± SEM (n = 4–6 mice). Means without a common letter differ, *P* < 0.05.

Further analysis of inflammatory markers revealed that hepatic expressions of the macrophage marker, F4/80^+^, and of monocyte/macrophage marker, CD68, were substantially decreased in the ethanol-fed WT mice and in the mLipin-1KO mice fed either with or without ethanol in comparison to WT controls, suggesting that ethanol feeding might increase apoptosis of F4/80^+^-positive Kupffer cells (Fig. 2C)^20^,^22^. Contrary to our previous results obtained with the Gao-binge ethanol feeding protocol^14^, hepatic gene expressions of the proinflammatory factors, tumor necrosis factor alpha (TNF-α) and Interleukin 6 (IL-6), were suppressed in the ethanol-fed WT mice and in the mLipin-1KO mice fed either with or without ethanol (Fig. 3A). These discrepancies could be resulted from differences in the genetic backgrounds of the mice (C57BL/6J and SV129^14^ vs. C57BL/6J^9^) and could be due to differences in their diet compositions (low fat^14^ vs. high-polyunsaturated fat^20^).

Interestingly, the serum levels of TNF-α and IL-6 were significantly elevated in the ethanol-fed WT mice compared to WT controls, suggesting that the ethanol-mediated increases in circulating TNFα and IL-6 might be largely derived from other organs (Fig. 3B,C). The serum levels of TNF-α and IL-6 were markedly reduced in the ethanol-fed mLipin-1KO mice compared with ethanol-fed WT mice (Fig. 3B,C).

We further examined gene expressions of M1 and M2 macrophage markers to check the status of macrophage polarization in livers of the ethanol-fed mLipin-1KO mice. Strikingly, mRNA gene expression of M1 marker Cd11c was nearly depleted whereas M2 marker Arg1 gene expression was mildly increased in livers of the ethanol-fed mLipin-1KO mice compared with all other groups (Fig. 3D).

As shown in Fig. 3E, the mRNA levels of IL-1β and NOD-like receptor family, pyrin domain containing 3 (NLRP3), two inflammasome components, were severely inhibited in the ethanol-fed WT and control diet-fed mLipin-1KO mice compared to WT controls. However, ethanol administration to the mLipin-1KO mice drastically decreased the expression of IL-1β, in parallel to a robust decrease in the mRNA expression of NLRP3 (Fig. 3E).

Hepatic gene expression of MIP-1α and MIP-1β, two potent chemoattractants for monocytes and/or neutrophils, were down-regulated in the mLipin-1KO mice fed either with or without ethanol compared to WT controls (Fig. 3F). Meanwhile, hepatic mRNA expression of chemokine MCP-1 was markedly reduced in the ethanol-fed mLipin-1KO compared to all other groups (Fig. 3F). Additionally, hepatic mRNA levels of adhesion molecules ICAM-1 and VCAM-1 were the lowest in the ethanol-fed mLipin-1KO mice compared to all other three groups (Fig. 3G). Collectively, these results clearly indicated that myeloid cell-specific lipin-1 deficiency drastically diminished inflammation in mice after ethanol administration.

### Myeloid cell-specific lipin-1 deficiency reduced circulating levels of lipocalin-2 (Lcn2) and serum amyloid A1 (Saa1) in the ethanol-fed mice

**Figure 4:**
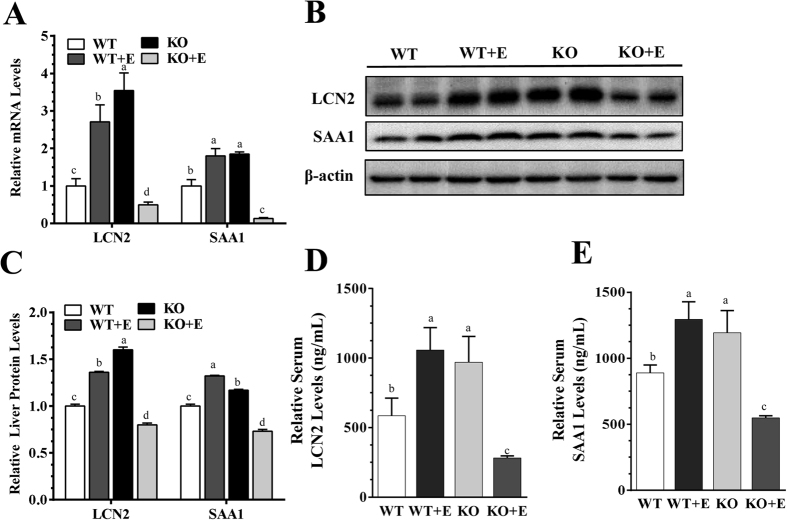
Myeloid cell-specific lipin-1 deficiency reduced circulating levels of lipocalin-2 (Lcn2) and serum amyloid A1 (Saa1) in mice after ethanol feeding. Wild-type (WT) or mLipin-1KO (KO) mice were pair-fed either a control diet or an ethanol-containing diet for 10 days followed by single gavage of ethanol. (**A**) Relative liver mRNA levels of Lcn2 and Saa1. (**B**) Representative Western analysis of liver Lcn2 and Saa1. (**C**) Relative liver protein levels of Lcn2 and Saa1. (**D**) Relative serum Lcn2 levels. (**E**) Relative serum Saa1 levels. Results are expressed as means ± SEM (n = 4–6 mice). Means without a common letter differ, *P* < 0.05.

We profiled expressions of Lcn2 and Saa-1, two major acute-phase molecules involved in regulating the ethanol-mediated inflammation process^9^,^14^,^21^. In comparison with the WT pair-fed control mice, Lcn2 mRNA and protein expression levels were significantly elevated in livers of the ethanol-fed WT mice, and the increases in Lcn2 gene expression were augmented in the control diet-fed mLipin-1KO mice (Fig. 4A–C). However, hepatic Lcn2 mRNA and protein levels in the ethanol-fed mLipin-1KO were drastically reduced to levels lower that of the WT controls (Fig. 4A–C). Accordingly, the serum levels of Lcn2 were significantly elevated in the ethanol-fed WT mice and control diet-fed mLipin-1KO mice to ∼180% and ∼167% of the WT controls, respectively (Fig. 4D). Strikingly, serum Lcn2 levels in the ethanol-fed mLipin-1KO mice were markedly reduced to ∼48% of WT controls (Fig. 4D).

While hepatic Saa1 gene and protein expression levels were similarly increased in the ethanol-fed WT and in control diet-fed mLipin-1KO mice compared to WT controls, ethanol feeding to the mLipin-1KO mice drastically decreased Saa1 expression (Fig. 4A–C). Consistently, serum Saa1 levels in the ethanol-fed WT mice and control diet-fed mLipin-1 mice were significantly elevated to ∼146% and ∼134% of WT controls, respectively (Fig. 4E). However, ethanol administration to mLipin-1KO mice robustly reduced circulating Saa1 to ∼62% of WT controls (Fig. 4E).

Taken together, these findings demonstrated that myeloid cell-specific lipin-1 deficiency markedly reduced expression and circulating levels of both Lcn2 and Saa1 in the ethanol-administrated mice.

### Myeloid cell-specific lipin-1 deficiency induced adipose-derived hormone adiponectin in the ethanol-fed mice

Altered adipose adiponectin production and its hepatic signaling have been implicated in the pathogenesis of ALD^17^,^23^. To understand underlying mechanism contributing to an attenuation of hepatic inflammation in the ethanol-fed mLipin-1KO mice, we examined adiponectin and its hepatic signaling.

**Figure 5:**
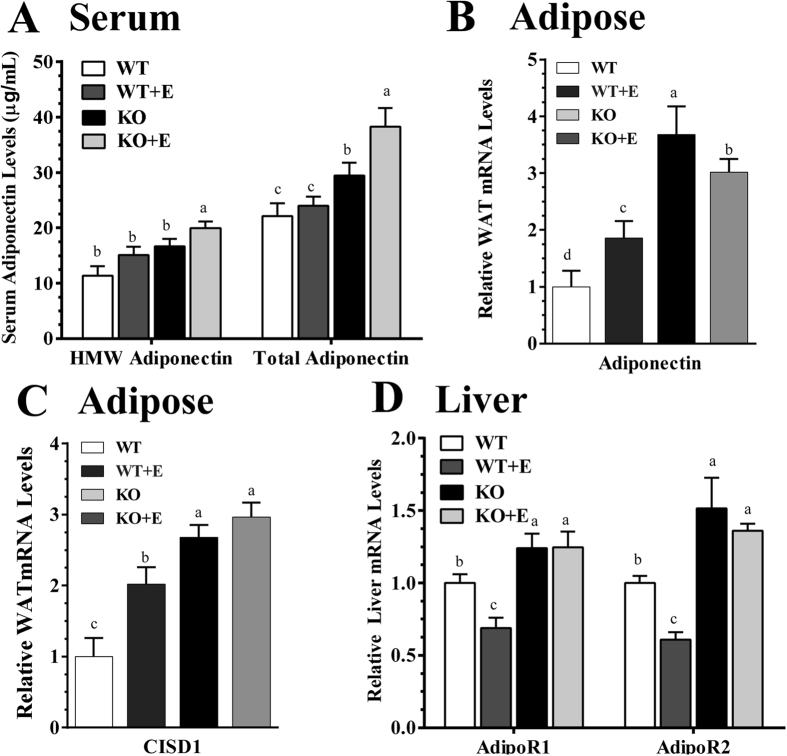
Myeloid cell-specific lipin-1 led to induction of adipose-derived hormone adiponectin in ethanol-administrated mice. Wild-type (WT) or mLipin-1KO (KO) mice were pair-fed either a control diet or an ethanol-containing diet for 10 days followed by single gavage of ethanol. (**A**) Serum protein levels of HMW and total adiponectin levels. (**B**) Relative adipose mRNA levels of adiponectin. (**C**) Relative adipose mRNA levels of CISD1. (**D**) Relative liver mRNA levels of AdipoR1 and AdipoR2. Results are expressed as means ± SEM (n = 4–6 mice). Means without a common letter differ, *P* < 0.05.

The serum levels of high-molecular weight (HMW) adiponectin and total adiponectin proteins were similar between the WT mice fed either with or without ethanol (Fig. 5A). However, ablation of myeloid cell-specific lipin-1 caused a mild increase in circulating total adiponectin and HMW concentrations in the control-diet fed mLipin-1KO mice (Fig. 5A). Strikingly, administration ethanol to mLipin-1KO mice led to prominent increases in circulating levels of HMW and total adiponectin to ∼176% and ∼173% of WT control, respectively (Fig. 5A).

Surprisingly, adipose adiponectin mRNA expression was significantly increased in the ethanol-fed WT mice compared to WT controls (Fig. 5B). We have previously found that ethanol feeding significantly decreased adipose adiponectin gene expression in mice^17^,^23^. This discrepancy may be due to the use of a Lieber-DeCarli chronic ethanol feeding protocol in those studies^17^,^23^. As shown in Fig. 5B, the increases in adipose adiponectin gene expression were further augmented in the mLipin-1KO mice fed either with or without ethanol.

CISD1 (also called MitoNEET) is a protein in the outer membrane of mitochondria and involves in regulating the expression, release and production of adiponectin in adipose tissues^24^. In comparison with the WT control mice, adipose mRNA levels of CISD1 were significantly increased in the ethanol-fed WT and the increases were augmented in the control diet-fed mLipin-1KO control mice (Fig. 5C). More importantly, the ethanol-fed mLipin-1KO mice exhibited the highest mRNA levels of CISD1, suggesting that increased CISD1 expression in the ethanol-fed mLipin-1KO mice might contribute to the upregulation of adipose adiponectin in these mice (Fig. 5C).

Adiponectin transduces its signaling via two major adiponectin receptors (AdipoRs), AdipoR1 and AdipoR2. In comparisons with the WT controls, ethanol feeding to WT mice significantly reduced mRNA expression of hepatic AdipoR1 and AdipoR2 (Fig. 5D). Interestingly, myeloid cell-specific lipin-1 deficiency substantially increased mRNAs of hepatic AdipoR1 and AdipoR2 in mice fed either with or without ethanol to levels higher than in the WT pair-fed control mice (Fig. 5D).

Taken together, our data demonstrated that myeloid cell-specific lipin-1 deficiency elevated circulating levels of HMW and total adiponectin and enhanced hepatic AdipoR1 and AdipoR2 gene expression, which in turn, would lead to stimulated hepatic adiponectin signaling in mice after ethanol administration.

### Myeloid cell-specific lipin-1 deficiency enhanced ileum FGF15 synthesis and elevated serum FGF15 levels in the ethanol-fed mice

Fibroblast growth factor 15 (*Fgf15*, the mouse ortholog of human *FGF19*) is produced in the intestine and regulates postprandial liver metabolism^25^,^26^. Emerging evidences suggest association and potential dialogues between the adipocyte-derived adiponectin and ileum FGF15/19^26^. As such, we investigated whether myeloid cell-specific lipin-1 deficiency affects ileum FGF15 and its hepatic receptors and their modifications by ethanol administration in mice.

**Figure 6:**
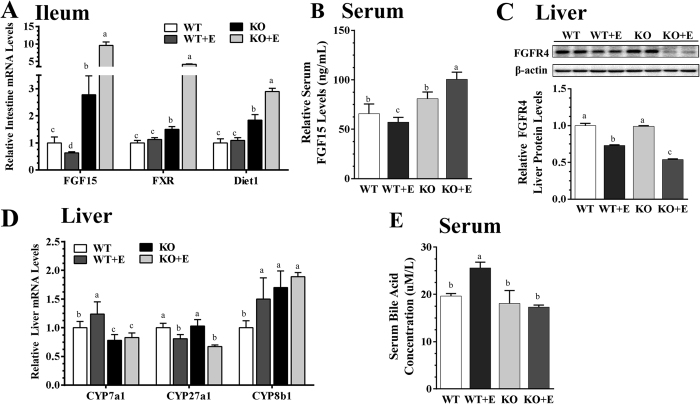
Myeloid cell-specific lipin-1 deficiency increased ileum FGF15 synthesis and normalized serum levels of bile acids in ethanol-fed mice. Wild-type (WT) or mLipin-1KO (KO) mice were pair-fed either a control diet or an ethanol-containing diet for 10 days followed by single gavage of ethanol. (**A**) Relative ileum mRNA levels of FGF15, FXR and Diet 1. (**B**) Serum levels of FGF15. (**C**) Protein levels of hepatic FGFR4. (**D**) Relative liver mRNA levels of Cyp7a1, Cyp27a1 and Cyp8b1. (**E**) Serum bile acid levels. Results are expressed as means ± SEM (n = 4–6 mice). Means without a common letter differ, *P* < 0.05.

The mRNA levels of ileum FGF15 were mildly but significantly reduced in the ethanol-fed WT mice compared to WT controls (Fig. 6A). However, in comparison with the WT controls, ablation of myeloid cell-specific lipin-1 significantly increased ileum FGF15 mRNAs (Fig. 6A). More strikingly, compared to the WT controls, ethanol administration to the mLipin-1KO mice robustly increased, up to ∼10-fold, the mRNA expression levels of ileum FGF15 (Fig. 6A).

Accordingly, the levels of serum FGF15 were significantly reduced to ∼86% in the ethanol-fed WT mice, but were enhanced to ∼122% in the control diet-fed mLipin-1KO mice, compared to WT control mice (Fig. 6B). More importantly, the robust increase of ileum FGF15 gene expression in the ethanol-fed mLipin-1KO mice coincided with a substantial increase in serum FGF15 levels to ∼152% of the WT controls (Fig. 6B).

As shown in Fig. 6A, although gene expression of intestine farnesoid X receptor (FXR) and Diet 1, two known molecules involved in regulating ileum FGF15/19 expression and production, were not affected by ethanol feeding to WT mice^27^. The mRNA expression of ileum FXR and Diet1 were mildly increased in the control diet-fed mLipin-1KO mice compared to WT controls. However, ethanol feeding to the mLipin-1KO mice robustly increased gene expressions of ileum FXR and Diet 1 compared to all other groups (Fig. 6A).

In hepatocytes, signaling of FGF15/19 requires the tyrosine kinase membrane receptor fibroblast growth factor receptor4 (FGFR4) and co-receptor β-Klotho (βKL)^25^,^26^. Protein levels of FGFR4 were reduced, as evidenced by Western Blot, in livers of the ethanol-fed WT compared to WT controls (Fig. 6C). Hepatic FGFR4 protein levels were markedly inhibited in the ethanol-fed mLipin-1 mice compared to all other three groups, consistent with the findings that induction of FGF15/19 from ileum was associated with liver FGFR4 deficiency (Fig. 6C).

Taken together, the data demonstrated that myeloid cell-specific lipin-1 deficiency increased ileum FGF15 synthesis and elevated circulating FGF15 levels in the ethanol-administrated mice.

### Myeloid cell-specific lipin-1 deficiency ameliorated the perturbation of bile acid homeostasis by ethanol administration in mice

Both adiponectin and FGF15/19 are capable of regulating bile acid hemostasis^25^,^26^,^28^,^29^. Hence, we examined whether hepatic myeloid cell-specific lipin-1 deficiency affects bile acid homeostasis.

Liver cholesterol 7α-hydroxylase (Cyp7a1), the rate-limiting enzyme for the “neutral” pathway of bile acid synthesis in liver, is partially suppressed by the gut-derived FGF15/19^25^. As shown in Fig. 6D, in comparison with the WT controls, mRNA expression of Cyp7a1 was significantly increased in livers of the ethanol-fed WT mice, confirming that the lower circulating levels of FGF15 promoted upregulation of hepatic Cyp7a1 expression. Furthermore, hepatic Cyp7a1 mRNAs were significantly repressed in the mLipin-1 mice fed with either a control diet or an ethanol-containing diet compared with WT controls, indicating that myeloid cell-specific lipin-1 deficiency-mediated elevation of ileum FGF15 transduced signals to livers of these mice (Fig. 6D).

Hepatic mRNA expression of Cyp27a1, which catalyzes the initial step of the alternative bile acid biosynthetic pathway, was significantly suppressed in the ethanol-fed WT mice and the suppression was slightly exacerbated in the ethanol-fed mLipin-1KO mice (Fig. 6D). Interestingly, mRNA level of Cyp8b1, a key enzyme controlling synthesis of cholic acid, was significantly elevated in the ethanol-fed WT mice, and in the mLipin-1KO mice fed either with or without ethanol compared to WT control mice (Fig. 6D).

Ethanol feeding to WT mice significantly increased serum bile acid to ∼130% of the WT controls, indicating dysregulated bile acid homeostasis in the ethanol-fed WT mice (Fig. 6E). Remarkably, serum levels of total bile acid in the ethanol-fed mLipin-1KO mice were reduced to ∼90% of WT controls (Fig. 6E).

### Myeloid cell-specific lipin-1 deficiency attenuated the NF-κB signaling activation by ethanol administration in mice

**Figure 7:**
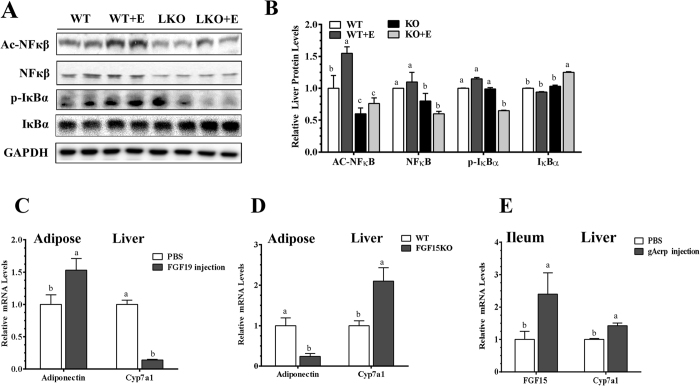
Myeloid cell-specific lipin-1 deficiency attenuated hepatic NF-kB activity in the ethanol administrated mice. Wild-type (WT) or mLipin-1KO (KO) mice were pair-fed either a control diet or an ethanol-containing diet for 10 days followed by single gavage of ethanol. (**A**) Representative Western analysis of liver Ac-NF-κB, NF-κB, p-IκBα and IκBα. (**B**) Relative liver protein levels of liver Ac-NF-κB, NF-κB, p-IκBα and IκBα. (**C**) Male C57/6J mice were treated by intraperitoneal injection 1 mg/kg mouse body weight of recombinant FGF19 for six hours. Relative adipose mRNA levels of adiponectin. Relative liver mRNA of Cyp7a1. (**D**) Male intestinal FGF15 knock out (FGF15KO) mice and LOX littermates (WT) mice were fed chow diets. Relative adipose mRNA levels of adiponectin. Relative liver mRNA levels of Cyp7a1. (**E**) Male C57/6J mice were treated by intraperitoneal injection with 0.5 μg/g mouse body weight of recombinant human globular adiponectin (gAcrp) for three days. Relative ileum FGF15. Relative liver mRNA of Cyp7a1. Results are expressed as means ± SEM (n = 4–6 mice). Means without a common letter differ, *P* < 0.05.

We investigated nuclear Factor Kappa B (NF-kB) signaling in the mechanism of attenuating hepatic inflammatory process by myeloid cell-specific lipin-1 deficiency. Ethanol feeding to WT mice stimulated NFκB activity, as revealed by increased levels of acetylated NF-κB and of phosphorylated IκBα and a lower level of IκBα level compared to the WT control mice (Fig. 7A,B). Activation of NF-κB, particularly the levels of p-IκBα, was markedly reduced in livers of the ethanol-fed mLipin-1KO mice compared to all other groups (Fig. 7A,B).

Collectively, these results demonstrated that myeloid cell-specific lipin-1 deficiency attenuated hepatic NF-κB activity and subsequently reduced inflammation and liver injury by ethanol in mice.

### Recombinant FGF19 inhibited Lcn2 and Saa1 gene expressions in cultured mouse AML-12 hepatocytes

We examined whether FGF15/FGF19 regulates Lcn2 and Saa1 gene expressions in cultured mouse AML-12 hepatocytes. Treatment with recombinant FGF19 for 24 hours significantly inhibited mRNA expressions of Lcn2 and Saa1 in a dose-dependent manner with a maximal effect at 50 ng/ml in AML-12 cells (Supplementary Fig. S1).

Furthermore, although Lcn2 mRNA levels were significantly increased by treatment with lipopolysaccharide (LPS) (100 ng/ml) or ethanol (50 mM), co-incubation with FGF19 (50 ng/ml) completely abolished the ability of LPS or ethanol to induce Lcn2 gene expression in AML-12 cells (Supplementary Fig. S1). The ability of LPS to induce Saa1 gene expression was also largely blunted by FGF19 treatment (Supplementary Fig. S1). Interestingly, co-incubation of FGF19 did not affect the ethanol-mediated induction of Saa1 mRNA abundance (Supplementary Fig. S1).

Collectively, these data demonstrated that recombinant FGF19 attenuated Lcn2 gene expression in AML-12 hepatocytes exposed to LPS or ethanol. Furthermore, FGF19 diminished the Saa1 gene expression stimulated by LPS but not by ethanol in AML-12 cells.

### Adiponectin and FGF19 regulated each other’s gene expression in mice

We evaluated whether FGF15/19 regulates adipose adiponectin expression by treating mice with recombinant FGF19. Adipose adiponectin mRNA expression levels were significantly increased and hepatic Cyp7a1 mRNA levels were suppressed (Fig. 7C) in the FGF19-treated mice compared to vehicle-injected control mice. More strikingly, in comparison to the WT control mice, FGF15 knockout (FGF15KO) mice displayed significantly lower adipose adiponectin mRNA abundance and higher hepatic Cyp7a1 mRNA expression (Fig. 7D).

To investigate the effect of adiponectin on ileum FGF15 synthesis, we treated mice with recombinant globular adiponectin (gAcrp), and observed significantly higher expression levels of ileum FGF15 mRNA compared to the controls (Fig. 7E). Paradoxically, hepatic Cyp7a1 mRNA levels were slightly increased in gAcrp-treated mice compared to controls, implying that gAcrp may also directly regulate hepatic Cyp7A1 gene expression (Fig. 7E)^28^,^29^.

Taken together, these observations suggest a reciprocal regulation of adiponectin and FGF15/19 gene expression in mice.

## Discussion

**Figure 8:**
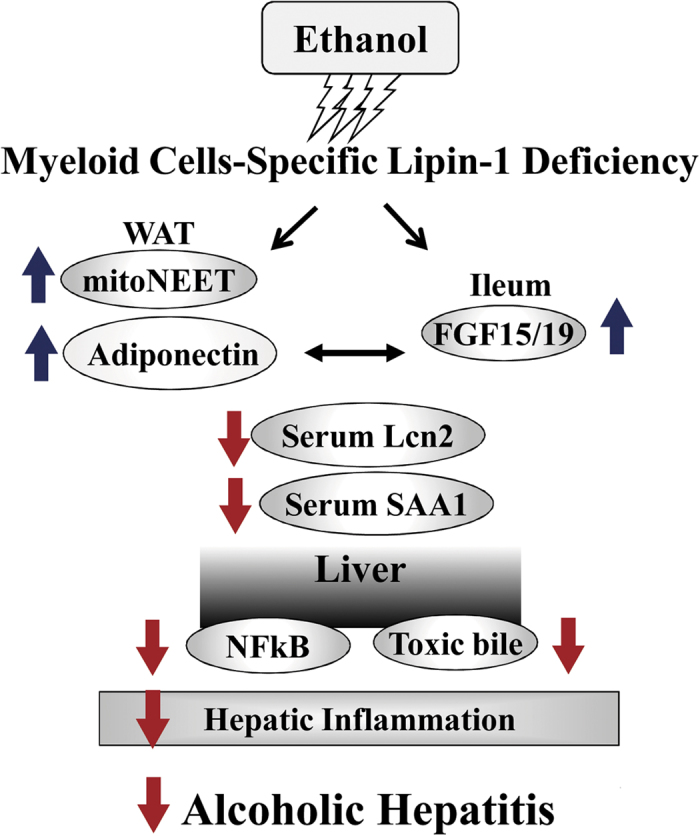
Proposed mechanisms of the protective action of myeloid cell-specific deficiency against experimental alcoholic hepatitis in mice. Following chronic-binge ethanol feeding, myeloid cell-specific lipin-1 deficiency induces the adipose-derived adiponectin and gut-derived FGF15 in mice. Elevated levels of adiponectin and FGF15 subsequently attenuate hepatic inflammation, and thus, alleviates experimental alcoholic hepatitis in mice.

In the present study, utilizing a mLipin-1KO mouse model, we unveiled an important role of myeloid cell-specific lipin-1 in experimental alcoholic steatohepatitis in mice. Myeloid cell-specific lipin-1 deficiency diminished hepatic inflammation, dramatically attenuated hepatic expression of a panel of inflammatory mediators, and alleviated the ethanol-induced liver injury in mice. Mechanistically, ethanol administration to mLipin-1KO mice increased gene expression and serum levels of the adipose-derived adiponectin and of ileum-derived FGF15. Through activating endocrine adiponectin-FGF15 axis, mLipin-1 deficiency normalized serum bile acid levels, inhibited hepatic NF-κB signaling, limited inflammation, and thus, protected liver from ethanol-induced damage. Altogether, our findings demonstrated that myeloid cell-specific deletion of lipin-1 ameliorated hepatic inflammation but not steatosis and alleviated liver injury in the ethanol-fed mice largely via induction of endocrine adiponectin-FGF15 axis and stimulation of adiponectin-FGF15 mediated hepatic signaling (Fig. 8).

In contrast to lipin-1 in the hepatocyte, which plays an anti-inflammatory role in the development and progression of alcoholic steatohepatitis in mice^9^, mLipin-1KO mice exhibited less hepatic inflammation and less liver injury but slightly worse steatosis following the ethanol administration. It is likely that lipin-1 regulates the inflammation process, and contributes to the development of alcoholic liver injury by cell-specific mechanisms. Two lipin-1 protein isoforms, lipin-1α (891 amino acids) and lipin-1β (924 amino acids), are derived from *Lpin 1* alternative mRNA splicing in liver^3^. Previously, we observed that lipin-1α but not lipin-1β exerted anti-inflammatory actions in macrophages exposed to ethanol or LPS^10^. Therefore, we speculate that genetic ablation of myeloid cell-specific lipin-1 may affect mainly lipin-1β but not lipin-1α. In this scenario, the diminished inflammatory responsiveness to ethanol in the mLipin-1KO mice could be due to a loss of lipin-1β function in myeloid cells.

The precise mechanisms underlying the interplay between adiponectin and FGF15 and how hepatic adiponectin-FGF15 signaling is affected by ethanol and myeloid cell-specific lipin-1 remain to be determined. Because both hormones are anti-inflammatory, they could exert their beneficial effects independently in the livers of ethanol-fed mLipin-1 mice^30^,^31^. On the other hand, adiponectin could be a critical driver of the liver improvements elicited by FGF15. Elevated FGF15 in the ethanol-fed mLipin-1 mice might also facilitate liver responses to adiponectin. Given that adiponectin and FGF15/19 share many signaling pathways and modulate common targets, adiponectin and FGF15 may also synergistically or additively exert anti-inflammatory effects and alleviate liver injury in the mLipin-1KO mice after ethanol administration. Additional ethanol feeding experiments using intestine-specific FGF15 knockout mice administrated with recombinant adiponectin or using adiponectin null mice treated with recombinant FGF15/19 should clarify the relative contributions of these endocrine hormones to the ethanol-mediated hepatic inflammatory process and alcoholic liver injury. The precise role of endocrine adiponectin-FGF15/19 axis in the context of experimental ALD in mice is currently under investigation in our laboratory.

Another important finding of the present study is that adiponectin and FGF15/19 regulate each other’s gene expression in mice, confirming an association and cross-talk between adipose adiponectin and ileum FGF15/19^26^. However, the precise mechanisms by which adiponectin and FGF15/19 affect each other’s gene expression and their secretion remain to be elucidated. In adipocytes, mitoNEET (CISD1) can regulate adiponectin both transcriptionally and post-transcriptionally^24^. Interestingly, we found that induction of mitoNEET and adiponectin gene expression in adipose tissues of the ethanol-fed mLipin-1KO mice shared a similar pattern. Thus, it is tempting to speculate that ileum FGF15 may upregulate adipose adiponectin via modulation of mitoNEET. The events and signaling pathways triggered by adiponectin or FGF15/19 to regulate each other’s gene expression and secretion warrants investigation. The precise cellular events by which myeloid cell-specific lipin-1 deficiency up-regulates hepatic AdipoR1/AdipoR2 levels remain to be investigated. AdipoR1/R2 expression are known to be inhibited by inflammatory signals, such as LPS and reactive oxygen species^32^,^33^. We have demonstrated that myeloid cell-specific lipin-1 deficiency elicits anti-inflammatory effects in mice. Therefore, it is logical to speculate that myeloid cell-specific lipin-1 deficiency may up-regulate hepatic AdipoR1/R2 via it’s anti-inflammatory properties in mice.

It is intriguing that deletion of lipin-1 in myeloid cells resulted in ileum FGF15 induction in the ethanol-fed mice. In intestine, both FXR and Diet 1 are major regulators of FGF15 synthesis and production^25^,^27^. Given that ethanol feeding to mLipin-1KO mice robustly increased gene expressions of FXR and Diet 1 in intestine, FXR and Diet 1 might directly involve in upregulating FGF15 in the mLipin-1KO mice in response to ethanol challenge. Furthermore, ileum FGF15/19 is known to be produced in response to bile acid absorption acting on the FXR^25^,^27^. Therefore, we speculate that myeloid cell-specific lipin-1 deficiency might induce bile acid absorption in ileum or modulate the intestinal synthesis of a specific bile acid which, in turn, lead to FGF15 induction in mice after ethanol administration. On the other hand, myeloid cell-specific lipin-1 deficiency might indirectly upregulate ileum FGF15 through enhanced adiponectin signaling in ethanol-fed mice.

Both Lcn2 and Saa-1 have recently been identified as major players in driving inflammation and causing development and progression of alcoholic steatohepatitis in rodents and human^9^,^14^,^21^,^34^. It is intriguing that the myeloid cell-specific lipin-1 deficiency drastically reduced the expressions and levels of Lcn2 and Saa1 in the ethanol administrated mice. Lcn2 plays an important role in promoting neutrophil infiltration in the pathogenesis of ALD^21^. The lower level of Lcn2 might be response for the attenuated neutrophil infiltration observed in the livers of ethanol-fed mLipin-1KO mice.

We provided evidences for the first time that the ileum-derived FGF19 repressed gene expressions of Lcn2 and Saa1 in cultured AML-12 hepatocytes. We have also observed that co-administration of recombinant adiponectin to the ethanol-fed mice abolished the ability of ethanol to up-regulate hepatic Lcn2 and Saa1 and alleviated alcoholic fatty liver injury in mice (M.Y., unpublished data). Therefore, we speculate an interplay between the adiponectin-FGF15/19 axis and Lcn2-Saa1 axis being coordinately regulated by ethanol and the myeloid cell-specific lipin-1. Giving that alcoholic liver injury is driven by organ cross-talks, it is also likely that adiponectin, FGF15/19, Lcn2 and Saa1 serve as crucial endocrine mediators in the cross-talks among adipose, intestine, and liver in the pathogenesis of ALD^1^,^2^,^3^.

In summary, our current findings suggest that reducing the myeloid cell-specific lipin-1 selectively attenuates the inflammatory responses and ameliorate liver damage by ethanol consumption in mice. More importantly, we provide novel mechanistic insights in the cooperative actions of adiponectin and FGF15 and their hepatic signaling in protecting liver from ethanol-induced injury in the mLipin-1KO mice. Further investigations are necessary before our current findings in mice can be translated to human. Several lines of evidence have suggested that ethanol-mediated dysregulation of lipin-1 function may contribute to the development and progression of human ALD^14^,^35^. Whether targeted modulation of myeloid cell-specific lipin-1 can provide therapeutic benefits in controlling the ethanol-induced inflammatory conditions in human should be the focus of future research. Our current findings also raise exciting possibilities of novel treatment strategies for human alcoholic hepatitis utilizing pharmacological modulation of the endocrine adiponectin-FGF15/19 axis.

## Methods

### Generation of myeloid cell-specific lipin-1 knockout (mLipin-1KO) mice

Lipin-1^loxP/loxP^ mice were generated as described previously^9^,^19^. Mice expressing Cre recombinase driven by the lysozyme promoter [Lysozyme-Cre mice, (B6.129P2-lyz2^tm1(cre)Ifo^/J)] were purchased from Jackson Laboratory (Bar Harbor, ME, USA). Lipin-1^flox/flox^ and LysM-Cre mice were crossed to generate myeloid cell-specific lipin-1 knockout mice (mLipin-1KO) mice (genetic background: C57BL/6 and SV129 background). Lipin-1^flox/flox^ Lysozyme-Cre^+^ (mLipin-1KO) and their age-matched Lox controls (WT) were used for the studies. All the animal experiments were approved by the Institutional Animal Care and Use Committee of Northeast Ohio Medical University (NEOMED). All the experiments were performed in accordance with the relevant guidelines and regulations.

### Ethanol feeding experiments

Liquid diets were based on the Lieber-DeCarli formulation and provided 1 kcal/ml (prepared by Frenchtown, New Jersey, USA). A mouse model of chronic plus single binge ethanol consumption (referred as Gao-binge model) was used as described^20^. Male mLipin-1KO mice and their age-matched littermate Lox controls (WT) older than ten to twelve weeks of age were divided into four dietary groups: (**1**) WT control; (**2**) WT plus ethanol (E; identical to the control diet but with 5% w/v ethanol added); (**3**) mLipin-1KO control; (**4**) mLipin-1KO plus ethanol. All mice were first fed a liquid control diet for 5 days. Ethanol groups were then fed a liquid diet containing 5% w/v ethanol for 10 days while control mice were pair-fed to their ethanol fed counterparts for 10 days. At day 11, mice in ethanol groups were given a single dose of ethanol gavage (5 g/kg body weight, 20% ethanol), while mice in WT or mLipin-1KO control groups were given an isocaloric dose of dextrin maltose gavage. The gavage was performed in the early morning, and mice had access to the diets after alcohol gavage. The mice were euthanized and blood and tissue samples were collected 9 h post-gavage. Liquid diets were freshly prepared from powder daily. For mice on an ethanol-containing diet, animal cages were placed on heating pads to maintain body temperature to compensate for potential alcohol-induced hypothermia. Food intake was recorded daily. During the entire feeding period, mLipin-1KO mice and their WT counterparts were observed to consume similar volumes of ethanol containing-diets.

### Recombinant adiponectin or recombinant FGF19 treatment in mice

10–13 week old male C57BL/6J mice were treated by intraperitoneal injection with 0.5 μg/g mouse body weight of recombinant human globular adiponectin (gAcrp) (Shenandoah Biotech, Warwick, PA) for three days in PBS or vehicle (PBS). 12 week old male C57BL/6J mice were treated by intraperitoneal injection with 1 mg/kg mouse body weight of recombinant FGF19 (Gold Biotech, St. Louis, MO) in PBS or vehicle (PBS) and then sacrificed six hours later. Then all the mice were euthanized, blood, liver, adipose tissue and terminal small intestine (ileum) samples were collected.

### FGF15 knockout mice (FGF15KO)

FGF15KO (FGF15KO) mice were kindly provided by Dr. Rohit Kohli (Cincinnati Children’s Hospital Medical Center). Those mice are on the C57BL/6J genetic background.

### Liver Histology

Liver tissues were fixed in 10% formalin and embedded in paraffin. Embedded liver tissues were sectioned and then stained with hematoxylin and eosin (H&E)^9^. Embedded liver tissues were also subjected to immunohistochemical staining for MPO by using rabbit anti-MPO polyclonal antibody (Abcam Inc, Cambridge, MA). Sections were incubated with Alexa Fluor 488 conjugated-goat anti-rabbit IgG secondary antibody (Thermo Fisher Scientific, Rockford, IL) for a MPO antibody. Positive cells for MPO were randomly photographed using a fluorescence microscope, and positive density was quantified using the software Image-Pro Plus (Media Cybernetics, Bethesda, MD).

### Biochemical analysis

Liver triglyceride cholesterol was measured as described previously^9^,^14^.

### ELISA Assays of animal Serum

Serum levels of ALT and AST were determined using the MaxDiscovery ALT (3460-08) and AST (5605-01) Assay Kits (Bioo-Scientific, Austin, TX). Plasma or tissue fatty acids, triglyceride and cholesterol were determined using the Infinity Cholesterol (TR13421) and Triglyceride (TR22421) Reagents (Thermo Scientific, Waltham, MA). Serum level of Lcn2 was measured by Lipocalin-2/ab199083 Mouse ELISA Kit (Abcam, Cambridge, MA). Serum level of Saa1 was measured by Saa1/ab157723 Mouse Elisa Kit (Abcam, Cambridge, MA). Serum level of FGF15 was measured by FGF15/LS-F11446 Mouse Elisa Kit (LifeSpan BioSciences, Inc, Seattle, WA). Serum levels of total adiponectin were determined using a commercial ELISA kit from BioVendor (Candler, NC). Serum levels of HMW forms of adiponectin were determined with Mouse Adiponectin Assay Kit from ALPCO # 47-ADPMS-E01 (Salem, NH). Concentrations of total bile acids in serum were determined using a 3α-hydroxysteroid dehydrogenase method (Diazyme). Analyses were performed with a SpectraMax i3x microplate reader (Molecular Devices, Sunnyvale, CA).

### Expression analyses

Liver, adipose tissues, and ileal samples were collected for mRNA analysis. The ileal samples were taken approximately 2 cm away from the ileo-cecal junction. Total RNA was extracted using the RNeasy kit (Qiagen) and cDNA was prepared using the Quantitech Reverse Transcription kit (Qiagen). Real-time quantitative PCR (qPCR) amplification was performed using the CFX96 Touch Real-Time PCR Detection System (Bio-Rad Laboratories) with FastStart Universal SYBR Green Master Mix with ROX (Roche Diagnostics)^9^,^14^. Relative mRNA levels were calculated using the comparative cycle threshold (Ct) method and were normalized to the values of glyceraldehyde-3-phosphate dehydrogenase (GAPDH) mRNA levels. The qPCR primers used in this study are listed in Table S1 (Supplementary Table S1).

Western blot analyses were performed by separating liver extracts by electrophoresis in 10% and/or AnyKd Mini-Protean TGX gels using Bio-Rad Mini-PROTEAN Tetra Cell System and transferred to PVDF membranes via Bio-Rad Blotting Module^9^,^14^. Multiple protein band quantification and analysis were performed using AlphaView software version 3.4 (ProteinSimple). SIRT1, NF-κB, acetylated NF-κB and GAPDH antibodies were purchased from Cell Signaling Technology (Danvers, MA). Lcn2, Saa1, and FGFR4 antibodies were purchased from Abcam (Cambridge, MA).

### Studies with Mouse AML-12 cells Hepatocytes

Mouse AML-12 hepatocytes (American Type Culture Collection, Manassas, VA) were cultured in DMEM/F12 medium supplemented with 10% fetal bovine serum (FBS), 100 μg/ml streptomycin, 63 μg/ml penicillin G, 0.1 μM dexamethasone, and insulin-transferrin-selenium (ITS; Gibco-BRL). For the treatment, hepatocytes were grown at 37 °C in an atmosphere of 5% CO_2_. At ∼80% confluence, the cells were then incubated overnight in a serum-free medium.

### Statistical analysis

All results are provided as means ± standard errors (SEM). Statistical analysis was performed using GraphPad Prism (San Diego, CA). The significance of differences between two groups was determined by unpaired two-tailed Student’s *t*-test. For comparison of multiple groups, one-way analysis of variance (ANOVA) test followed by the post-hoc Bonferroni test was applied. Significance was accepted at a *p* value of <0.05.

## Additional Information

**How to cite this article**: Wang, J. *et al.* Myeloid Cell-Specific Lipin-1 Deficiency Stimulates Endocrine Adiponectin-FGF15 Axis and Ameliorates Ethanol-Induced Liver Injury in Mice. *Sci. Rep.* **6**, 34117; doi: 10.1038/srep34117 (2016).

## Supplementary Material

Supplementary Information
