# Supplementary material for: Myeloid Cell-Specific Lipin-1 Deficiency Stimulates Endocrine Adiponectin-FGF15 Axis and Ameliorates Ethanol-Induced Liver Injury in Mice
Source: Sci Rep. 2016 Sep 26;6:34117. doi: 10.1038/srep34117 (PMC5036185; doi:10.1038/srep34117)
Supplement: Supplementary Information [file srep34117-s1.pdf]

Supplementary Information

Myeloid Cell-Specific Lipin-1 Deficiency Stimulates Endocrine Adiponectin-FGF15 Axis and Ameliorates Ethanol-Induced Liver Injury in Mice

Jiayou Wang[1], Chun-ki Kim[1], Alvin Jogasuria[1], Yoonhee Han[1], Xudong Hu [1,2], Jiashin Wu[1], Hong Shen[1,3], Roman Chrast[4], Brian N. Finck[5], and Min You[1*]

[1]Department of Pharmaceutical Sciences, College of Pharmacy, Northeast Ohio Medical University, Rootstown, OH 44272, USA

[2]Department of Biology, School of Basic Medical Science, Shanghai University of Traditional Chinese Medicine, Shanghai, China.

[3]Department of Liver Diseases, Guangdong Hospital of Traditional Chinese Medicine in Zhuhai, Zhuhai, China

[4]Department of Neuroscience, Karolinska Institute, Stockholm, Sweden

[5]Division of Geriatrics and Nutritional Science, Washington University School of Medicine, St. Louis, USA

Running title: Lipin-1 and Alcoholic Hepatitis

Abbreviations used in the paper: ALD, alcoholic liver disease; ALT, alanine aminotransferase; AST, aspartate aminotransferase; AdipoR, adiponectin receptor; Cyp7a1, cholesterol 7α-hydroxylase; FGF, fibroblast growth factor; FGFR4, fibroblast growth factor receptor 4; FXR, farnesoid x receptor; gAcrp, recombinant globular adiponectin; HMW, high-molecular weight; KL, Klotho; Lcn2, Lipocalin 2; LPS, lipopolysaccharide; IL-1β, IL-6, Interleukin 6; MPO, myeloperoxidase; NF-κB, nuclear Factor Kappa B; NLRP3, NOD-like receptor family, pyrin domain containing 3; PAP, phosphatidate phosphohydrolase; SAA1, serum amyloid A-1; TLR, Toll-Like-Receptor; TNF-α, tumor necrosis factor-alpha; TG, triglyceride; WT, wild-type.

Table S 1

**Gene Abbreviations**	**Direction**	**Sequence (5'→3')**
F4/80+	Fwd	TGACTCACCTTGTGGTCCTAA
	Rev	CTTCCCAGAATCCAGTCTTTCC
CD68	Fwd	TGTCTGATCTTGCTAGGACCG
	Rev	GAGAGTAACGGCCTTTTTGTGA
Ly6G	Fwd	CTTCTCTGATGGATTTTGCGTTG
	Rev	AGTAGTGGGGCAGATGGGAAG
ICAM-1	Fwd	CAATTTCTCATGCCGCACAG
	Rev	AGCTGGAAGATCGAAAGTCCG
VCAM-1	Fwd	TGAACCCAAACAGAGGCAGAGT
	Rev	GGTATCCCATCACTTGAGCAGG
CD11C	Fwd	CTGGATAGCCTTTCTTCTGCTG
	Rev	GCACACTGTGTCCGAACTCA
Arg1	Fwd	CTCCAAGCCAAAGTCCTTAGAG
	Rev	AGGAGCTGTCATTAGGGACATC
MCP-1	Fwd	TTAAAAACCTGGATCGGAACCAA
	Rev	GCATTAGCTTCAGATTTACGGGT
MIP-1α	Fwd	TGAGAGTCTTGGAGGCAGCGA
	Rev	TGTGGCTACTTGGCAGCAAACA
MIP-1β	Fwd	AACACCATGAAGCTCTGCGT
	Rev	AGAAACAGCAGGAAGTGGGA
LCN2	Fwd	TGGCCCTGAGTGTCATGTG
	Rev	CTCTTGTAGCTCATAGATGGTGC
SAA1	Fwd	ACCTGTGGTTCGGAGGATGAA
	Rev	CCCAGACTCTGCAAATCTCATTC
Adiponectin	Fwd	AGGTTGGATGGCAGGC

	Rev	GTCTCACCCTTAGGACCAAGAA
CISD1	Fwd	GCTGTGCGAGTTGAGTGGAT
	Rev	TGGTGCGATTCTCTTTAGCGTA
AdipoR1	Fwd	TCCCAGGAACACTCCTGCTC
	Rev	CTTCTACTGCTCCCCACAGC
AdipoR2	Fwd	AGCCTCTATATCACCGGAGCTG
	Rev	GCTGATGAGAGTGAAACCAGATGT
FGF15	Fwd	ATGGCGAGAAAGTGGAACGG
	Rev	CTGACACAGACTGGGATTGCT
FXR	Fwd	GGCAGAATCTGGATTTGGAATCG
	Rev	GCTGAACTTGAGGAAACGGG
Diet1	Fwd	ACACTGGTTGGACCTATGCG
	Rev	TCAGCCTCGAATGCAACCTT
β-Klotho	Fwd	TGTTCTGCTGCGAGCTGTTAC
	Rev	CCGGACTCACGTACTGTTTTT
Cyp7a1	Fwd	GGGATTGCTGTGGTAGTGAGC
	Rev	GGTATGGAATCAACCCGTTGTC
Cyp27a1	Fwd	CCAGGCACAGGAGAGTACG
	Rev	GGGCAAGTGCAGCACATAG
Cyp8b1	Fwd	CCTCTGGACAAGGGTTTTGTG
	Rev	GCACCGTGAAGACATCCCC
GAPDH	Fwd	TGACCTCAACTACATGGTCTACA
	Rev	CTTCCCATTCTCGGCCTTG

Supplementary Figure Legend

Supplementary Figure S1: FGF19 regulated Lcn2 and SAA-1 gene expression in mouse AML-12 hepatocytes. (**A**) AML-12 hepatocytes were starved in serum-free medium overnight, and recombinant FGF19 was added at the indicated concentrations for 24 h. The mRNA expression of LCN2 or SAA1 was measured by qRT-PCR and normalized to GAPDH mRNA. (**B**) Starved AML-12 hepatocytes were cultured with either ethanol (50 mM; 24 h) or LPS (100 ng/ml, 6h) in the absence or presence of recombinant FGF19 (50 ng/ml, 24 h). The mRNA expression of the Lcn2 and SAA1 was normalized to GAPDH mRNA. Data are means±SEM for 3 replications. Means without a common letter differ, $P < 0.05$.

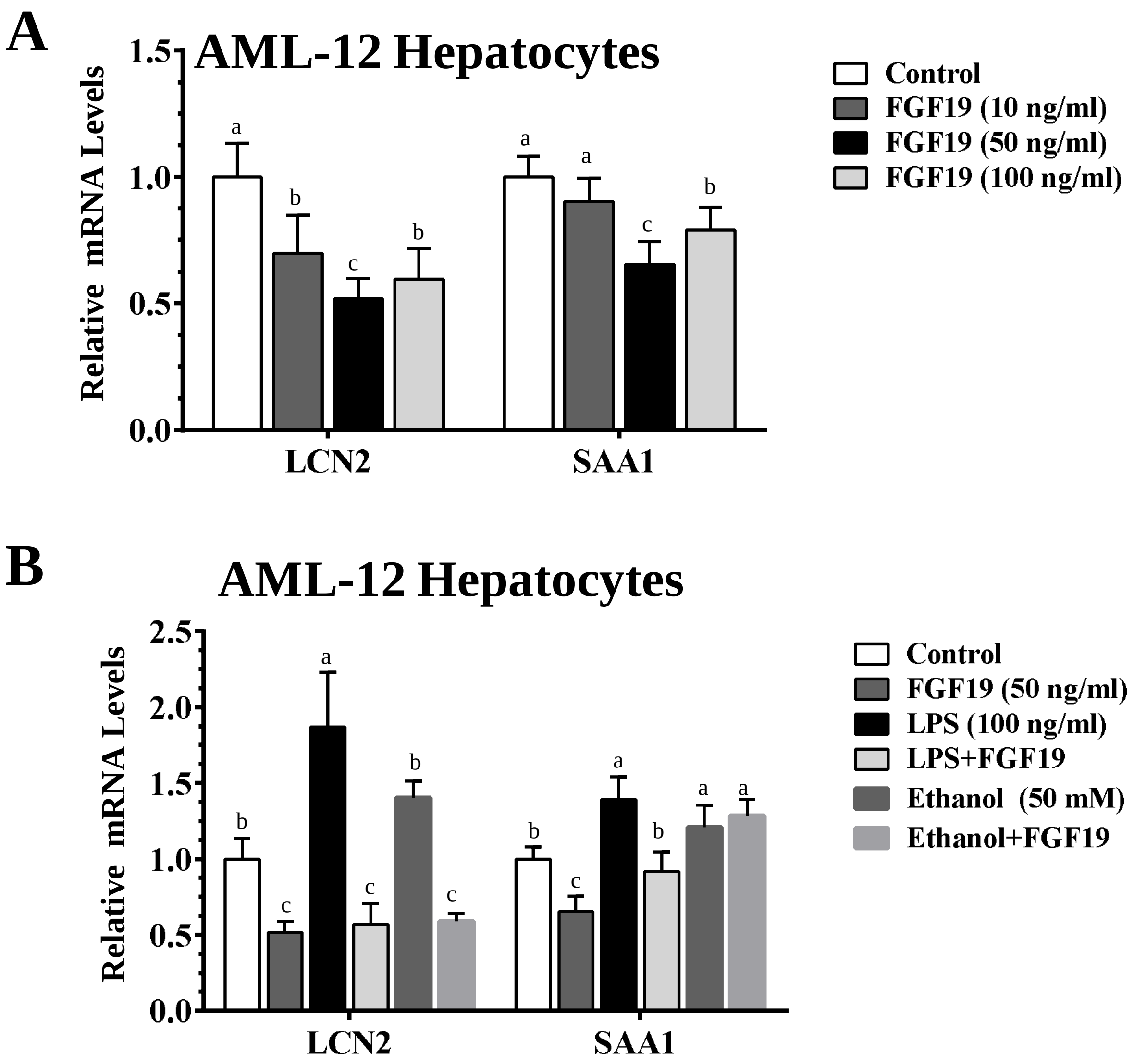

Supplementary Figure S1
